# Editorial: Interconnected impacts: climate change, biodiversity loss, and health

**DOI:** 10.3389/fpubh.2026.1912102

**Published:** 2026-07-08

**Authors:** Matilda van den Bosch, Inês Paciência, Wael K. Al-Delaimy, Jouni J. K. Jaakkola

**Affiliations:** 1 Institute for Global Health (ISGlobal), Barcelona, Spain; 2 Universitat Pompeu Fabra (UPF), Barcelona, Spain; 3 CIBER Epidemiología y Salud Pública (CIBERESP), Madrid, Spain; 4 European Forest Institute, Biocities Facility, Rome, Italy; 5 School of Population and Public Health, University of British Columbia, Vancouver, BC, Canada; 6 Center for Environmental and Respiratory Health Research (CERH), Research Unit of Population Health, University of Oulu, Oulu, Finland; 7 Herbert Wertheim School of Public Health and Human Longevity Science, University of California San Diego, La Jolla, CA, United States

**Keywords:** Anthropocene, food security, mental health, microbiome dynamics, nature connection, planetary health, respiratory health

**Figure 1:**
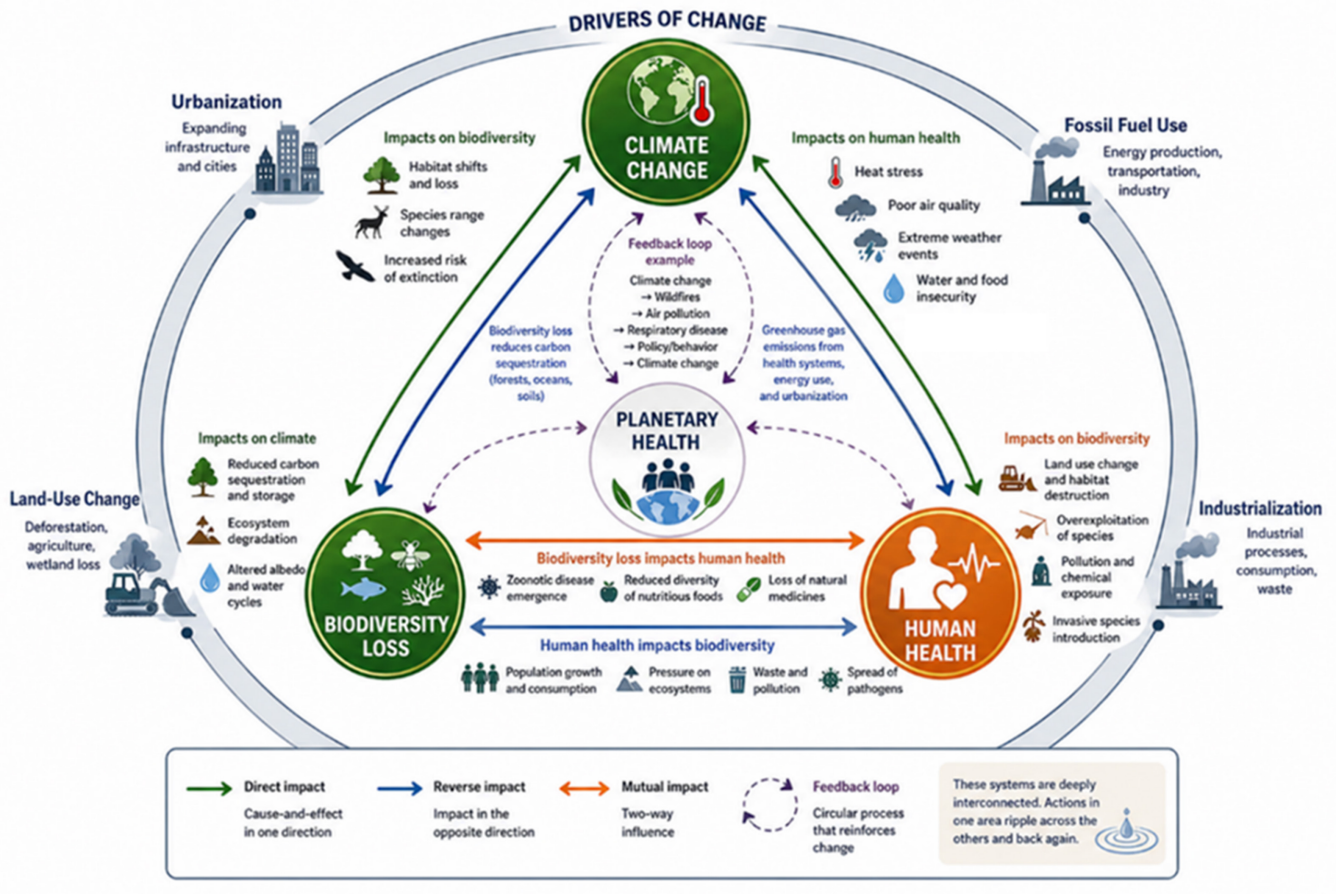
Interconnected impacts: climate change, biodiversity loss, and human health. Climate change, biodiversity loss, and human health are increasingly recognized as interconnected dimensions of a single global crisis. As illustrated, these systems are linked through bidirectional pathways and reinforcing feedback loops, driven largely by human activities such as fossil fuel use, industrialization, land-use change, and urbanization. The figure was developed with assistance from ChatGPT (OpenAI) and refined by the authors.

Climate change and biodiversity loss are increasingly recognized as interconnected global crises with profound implications for human health (1, 2). Historically, these challenges have often been addressed separately within environmental, ecological, and public health research. However, growing evidence demonstrates that climate-related environmental change, ecosystem degradation, and biodiversity decline interact through complex pathways that shape human exposure patterns, food systems, infectious and non-communicable diseases, mental health, and social vulnerability (3). The emerging planetary health framework emphasizes that human health is inseparable from the integrity of Earth's natural systems, calling for integrated and interdisciplinary approaches to research, prevention, and policy (4) (Figure 1).

This Research Topic, “*Interconnected impacts: climate change, biodiversity loss, and health*,” brings together contributions that collectively illustrate the diverse mechanisms through which environmental change affects health across ecological, biological, social, and psychological dimensions. The articles span multiple geographical settings and methodological traditions, including epidemiology, environmental health, participatory research, microbiome science, mixed methods research, and computational approaches. Together, they highlight both the complexity and urgency of addressing the health consequences of environmental degradation in a rapidly changing world.

Several contributions focus on how climate-related ecological disruption affects physical health through environmental exposures and altered ecosystem processes. In Senegal, Barclay-Derman et al. examine how environmental change influences food security and livelihoods in the Casamance region through a community-based participatory research approach. Their findings reveal that local communities perceive environmental degradation, including decreasing and irregular rainfall, deforestation, salinization, and declining river and marine health, as deeply interconnected with food insecurity, land abandonment, and cultural disruption. Importantly, the study demonstrates the value of integrating community knowledge and lived experience into climate adaptation and food system resilience strategies.

Complementing this perspective, Godoy-Vitorino et al. explore the intersection of environmental exposures, microbiome dynamics, and chronic disease risk in Puerto Rico, a climate-vulnerable region facing multiple environmental stressors. Their interdisciplinary perspective highlights how air pollution, fungal bioaerosols, Saharan dust events, and ecological disturbances may influence respiratory disease, cancer risk, immune regulation, and microbiome composition. The study underscores the importance of considering environmental microbiomes and broader ecological processes within environmental health research, particularly in regions disproportionately affected by climate vulnerability and infrastructural inequities.

The burden of respiratory disease in the context of biodiversity loss and climate change is further addressed by Deng, who presents a computational framework integrating deep learning and causal mapping approaches to assess climate-induced biodiversity loss and respiratory health outcomes. The author used a tailored analytical system by integrating GeoExposureNet and Causal-Aware Adaptive Mapping (CAM). Although methodologically distinct from the other contributions by heavily relying on publicly available data with all its limitations, this work reflects the growing importance of advanced analytical and data science approaches for understanding complex exposure–health relationships in environmental health research.

The Research Topic also highlights the broader systemic implications of environmental degradation for non-communicable diseases (NCDs) and public health through the lens of planetary health. Kakaraparthi et al. discuss the convergence of planetary health and NCDs, emphasizing how pollution, urbanization, food systems, and ecological degradation contribute to rising burdens of cardiovascular disease, respiratory illness, diabetes, and cancer. Their contribution reinforces the need to move beyond viewing NCDs solely as lifestyle-related conditions and instead recognize the critical role of environmental determinants and ecological sustainability in shaping chronic disease patterns globally. The health system, environmental, and behavioral interventions are proposed through policy solutions.

The collection also addresses the often-overlooked relationship between biodiversity, urban nature, and mental wellbeing. Schönbach et al. investigate the needs required to foster urban nature connectedness among female university students in Southern Germany using a mixed methods concept mapping approach. Their findings suggest that accessibility and situational factors play a central role in enabling connection with urban nature, with implications for urban planning, public health, and health equity. As urbanization continues globally, understanding how urban environments can support both mental health and human–nature relationships become increasingly important. This study also illustrates how biodiversity and access to green space may function as co-benefits for both planetary and human health.

Across these contributions, several common themes emerge. First, environmental change affects health through multiple intertwined pathways, including altered food systems, air quality, ecosystem services, microbial exposures, psychosocial processes, and chronic disease risks. Second, vulnerability is unevenly distributed, with disproportionate impacts on populations already facing social, economic, geographic, or infrastructural disadvantages. Third, the papers collectively demonstrate the importance of interdisciplinary and transdisciplinary approaches that bridge environmental science, public health, ecology, social science, urban planning, and data science.

Another recurring theme is the importance of moving beyond purely technocratic or reductionist understandings of environmental health. Several papers emphasize place-based knowledge, lived experience, social context, and cultural dimensions of environmental change. These perspectives are essential for developing equitable and context-sensitive adaptation and mitigation strategies that strengthen both ecological and human resilience.

It is important to note, that all these perspectives have to take into consideration the anthropogenic origin of climate change and biodiversity loss. At the core of the Anthropocene is the ongoing damage to the planet's sustainability. Biodiversity is a litmus test of the how well the planet is coping with the ongoing assault by humans on earth systems, which is impacting the health of humans now and in the future.

Taken together, this Research Topic highlights the need for integrated approaches capable of addressing the interconnected crises of climate change, biodiversity loss, and health. Protecting biodiversity and ecological integrity is not only an environmental imperative but also a public health necessity. Future research and policy efforts should continue to strengthen interdisciplinary collaboration, incorporate community perspectives, and develop prevention-oriented strategies that recognize the fundamental interdependence between healthy ecosystems and healthy populations.
